# cGAS Restricts PRRSV Replication by Sensing the mtDNA to Increase the cGAMP Activity

**DOI:** 10.3389/fimmu.2022.887054

**Published:** 2022-04-26

**Authors:** Xiao-Na Liu, Li-Wei Li, Fei Gao, Yi-Feng Jiang, Wan-Zhe Yuan, Guo-Xin Li, Ling-Xue Yu, Yan-Jun Zhou, Guang-Zhi Tong, Kuan Zhao

**Affiliations:** ^1^College of Veterinary Medicine, Hebei Agricultural University, Baoding, China; ^2^Shanghai Veterinary Research Institute, Chinese Academy of Agricultural Sciences, Shanghai, China; ^3^Hebei Veterinary Biotechnology Innovation Center , Hebei Agricultural University, Baoding, China

**Keywords:** PRRSV, cGAS, mtDNA, cGAMP, replication, antiviral activity

## Abstract

Porcine reproductive and respiratory syndrome virus (PRRSV) is an RNA virus that causes great economic losses globally to the swine industry. Innate immune RNA receptors mainly sense it during infection. As a DNA sensor, cyclic GMP-AMP synthase (cGAS) plays an important role in sensing cytosolic DNA and activating innate immunity to induce IFN-I and establish an antiviral cellular state. In contrast, the role of innate immune DNA sensors during PRRSV infection has not been elucidated. In this study, we found that cGAS facilitates the production of IFN-β during PRRSV infection. Western blot and virus titer assays suggested that cGAS overexpression suppressed the replication of multiple PRRSV strains, while knockout of cGAS increased viral titer and nucleocapsid protein expression. Besides, our results indicated that the mitochondria were damaged during PRRSV infection and leaked mitochondrial DNA (mtDNA) into the cytoplasm. The mtDNA in the cytoplasm co-localizes with the cGAS, and the cGAMP activity was increased when the cGAS was overexpressed during PRRSV infection. Furthermore, the cGAMP also possesses an anti-PRRSV effect. These results indicate for the first time that cGAS restricts PRRSV replication by sensing the mtDNA in the cytoplasm to increase cGAMP activity, which not only explains the molecular mechanism by which cGAS inhibits PRRSV replication but also provides research ideas for studying the role of the cGAS-STING signaling pathway in the process of RNA virus infection.

## Introduction

Porcine reproductive and respiratory syndrome (PRRS), characterized by manifest reproductive problems in sows and respiratory problems in piglets, is one of the most problematic infectious diseases for the swine industry worldwide (1). Since 2006, a highly-pathogenic form of the PRRS virus (HP-PRRSV) has circulated in China, resulting in considerable economic loss (2).

PRRSV, the causative agent of PRRS, is in the family *Arteriviridae* of the order *Nidovirales* (3). PRRSV is an enveloped, single-stranded, positive-sense RNA virus. Its genome RNA is approximately 15.4 kb consisting of at least 10 open reading frames (ORFs), a 5’-untranslated region (UTR), and a 3’-UTR (4). PRRSV was found to suppress interferon (IFN) production activated by double-stranded RNA (dsRNA). PRRSV proteins, namely, Nsp1, Nsp2, Nsp4, Nsp11, and N, have been identified and characterized as IFN antagonists (5, 6). To further ensure the normal physiological state of the host, the host has evolved a series of strategies to resist the virus. Immediately after PRRSV infection, the main host pattern recognition receptors (PRRs) are retinoic acid-inducible gene I (RIG-I), which can directly recognize and bind viral 5′-PPP RNA and short double-stranded RNA and then activate innate immunity against viral infections (7). So far, many interferon-stimulated genes (ISGs) that result in PRRSV resistance have been identified (8, 9).

Cyclic GMP-AMP (cGAMP) synthase (cGAS) is a newly identified DNA sensor that triggers IFN-I production. It plays an important role in sensing cytosolic DNA and triggering a stimulator of IFN genes (STING) dependent signaling to induce IFN-I (10). Additionally, PRR ligands, namely, bacteria, DNA viruses, and retroviruses, induce the expression of cGAS in an IFN-I-dependent manner. Besides, cGAS can positively regulate the production of IFN, so cGAS was considered a new ISG (11). During DNA virus infection, the virus genome is directly recognized and bound to cGAS to activate the cGAMP activity, which induces the production of IFN. Numerous DNA viruses activate the cGAS–STING pathway, and cGAS-deficient mice are more susceptible to lethal infection after exposure to many DNA viruses, namely, herpes simplex virus 1 (HSV-1), vaccinia virus (VACV), and murine gammaherpesvirus 68 (MHV68) (12). However, emerging evidence indicates that, in addition to its well-established role in sensing cytosolic DNA, the cGAS is also involved in restricting RNA virus infection, suggesting crosstalk exists between the innate sensing of cytosolic DNA and RNA (13). Several studies reveal that the replication of RNA viruses, namely, equine arterivirus (EAV), dengue virus (DENV), West Nile virus (WNV), influenza A virus (IAV), and chikungunya virus (CHIKV), is greatly facilitated in cells and mice with a deficiency of cGAS (14–16). Interestingly, we found that overexpressing cGAS inhibits PRRSV replication and knockout of cGAS increases PRRSV replication. However, PRRSV and other RNA viruses do not form dsDNA or DNA : RNA complexes during infection, so cGAS must be activated by other means during the infection of these RNA viruses, but the mechanism is unclear.

Here, we show that cGAS plays an important role in inhibiting PRRSV replication. PRRSV infection causes mitochondrial damage, resulting in mitochondrial DNA (mtDNA) leaking into the cytoplasm. cGAS is activated by binding to mtDNA in the cytoplasm, increasing cGAMP activity, promoting IFN production, and inhibiting PRRSV replication. Together, this study elucidates for the first time the molecular mechanism by which cGAS inhibits PRRSV replication and will provide research ideas for studying the role of the cGAS-STING signaling pathway in the process of RNA virus infection.

## Materials and Methods

### Cells and Virus

African green monkey kidney (Marc-145) cells were maintained in Dulbecco’s modified Eagle’s medium (DMEM) containing 10% fetal bovine serum (FBS; Gibco, Thermo Fisher Scientific, Waltham, MA, USA) at 37°C and under 5% CO_2_. The HP-PRRSV strain HuN4 (GenBank accession No. EF635006), a classic type 2 strain (vAPRRS; GenBank accession No. GQ330474), and a classic type 1 strain (vSHE; GenBank accession No. GQ461593). All the viruses, cells and vectors used in our experiment are stored in our lab.

### Construction of Plasmids

To create p3xFlag-cGAS, the porcine cGAS (MB21D1) gene (GenBank accession no. XM_013985148.2) was amplified from the porcine alveolar macrophages (PAMs) and cloned into p3xFlag. According to the instructions of the manufacturer, the plasmids were constructed by homologous recombination with the NEBuilder^®^ HiFi DNA Assembly Master Mix (New England Biolabs; Ipswich, MA). The primers used for gene amplification are listed in **Table 1**.

**Table 1:** Sequences of primers and gRNAs used in this study.

Primer	Sequence (5′−3′)
Monkey-IFN-β-F	GCAATTGAATGGAAGGCTTGA
Monkey-IFN-β-R	CAGCGTCCTCCTTCTGGAACT
β-actin-F	CGGGAAATCGTGCGTGAC
β-actin-F	ATGCCCAGGAAGGAAGGTTG
p3xFlag-cGAS-F	AAGCTTGCGGCCGCGATGGCGGCCCGGCGGGGAAAG
p3xFlag-cGAS-R	AGATCTATCGATGAATTTCACCAAAAAACTGGAAATCCA
cGAS-Exon1-gRNA-F	CACCGAGACTCGTTGCGGTCGGTC
cGAS-Exon1-gRNA-R	AAACGACCGACCGCAACGAGTCTC
cGAS-Exon2-gRNA-F	CACCGCTAGAAGAATATTCTGACAC
cGAS-Exon2-gRNA-F	AAACGTGTCAGAATATTCTTCTAGC

### Plasmid Transfection and Virus Challenge

Marc-145 cells cultured in 6-well plates were transfected with 1 μg of p3xFlag-cGAS using X-treme GENE HP DNA reagent (Roche Applied Science, Penzberg, Germany), to investigate the effect of cGAS on IFN-β production and PRRSV replication. Next, 24 h post-transfection (hpt), the cells were infected with PRRSV HuN4 (a multiplicity of infection, MOI, of 0.1). After inoculation for 1 h at 37°C, the supernatants were discarded, and the cells were washed three times with phosphate-buffered saline (PBS). The supernatant was harvested at 12, 24, 36, and 48 h post-infection (hpi), and the cells were lysed using RIPA lysis buffer (Thermo Fisher Scientific). IFN-β was assessed by quantitative real-time reverse-transcription polymerase chain reaction (qRT-PCR) and ELISA with the Monkey IFN-β ELISA Kit (Cusabio) according to the instructions of the manufacturer. Viral titers in the supernatants were determined using a microtitration assay according to the method of Reed and Muench (17). The amount of N protein was then detected in cell lysates by western blot (WB) using a mouse anti-N polyclonal antibody (1:1,000) produced by the authors.

### qRT-PCR

Total RNA was extracted using an RNeasy mini kit (Qiagen, Hilden, Germany), following the instructions of the manufacturer. Reverse transcription reactions were performed at 25°C for 5 min and 42°C for 1 h using the M-MLV reverse transcription-polymerase system (TaKaRa, Dalian, China). SYBR Premix Ex Taq™ (Takara) was used to quantify the levels of IFN-β. Relative expression levels were analyzed using the ΔΔCt method (18), with β-actin mRNA as a control. Primers are listed in **Table 1**. qPCR detected the mtDNA in the cytoplasm of Marc-145 cells after being infected with PRRSV (refer to 19).

### WB

Cell lysates were by incubating cells in RIPA for 15 min at 4°C with 1 mM phenylmethylsulfonyl fluoride and 1 mg/ml of protease inhibitor cocktail (Roche). After centrifuging at 12,000×*g* for 10 min, the supernatants of cell lysates were mixed with 5× sodium dodecyl sulfate-polyacrylamide gel electrophoresis (SDS-PAGE) sample loading buffer (Beyotime) and placed in boiling water for 5 min. The proteins were separated by SDS-PAGE and transfected onto a nitrocellulose membrane. The membrane was blocked in 5% skim milk for 2 h at room temperature before incubating with the indicated antibody for 1 h at room temperature. After 3 washes with Tris-buffered saline with 0.1% Tween 20, the membrane was incubated for 1 h at room temperature with horseradish peroxidase-conjugated goat anti-mouse/rabbit IgG (H + L) (1:5,000). The membranes were visualized by treating them with Pierce ECL WB substrate (Thermo Fisher Scientific). For the quantification of target proteins, their levels were normalized to the levels of β-actin.

### Cytoplasmic Mitochondrial DNA Extraction

Marc-145 cells were seeded in a 10-cm dish and infected with PRRSV at different MOIs (0.5, 1, and 1.5). The uninfected cells act as a control. At 24 hpi, the culture medium was discarded, and the cells were washed twice with PBS. The cells were scraped off with the scraper and then sent to separate the cytoplasm without mitochondria with the Mitochondria Isolation Kit for Cultured Cells kit (Thermo Fisher). The cytoplasm DNA was extracted with a QIAamp DNA Mini Kit (QIAGEN) and quantified with qPCR.

### Establishment of the cGAS Knockout (cGAS-KO) Marc-145 Cells

The cGAS-KO Marc-145 cells were generated using the CRISPR/Cas9 system and then used to evaluate the effect of cGAS deletion on PRRSV replication. Two guide RNAs, RNA1 and RNA2, were designed by the online-Optimized CRISPR Design tool (http://crispr.mit.edu/) (**Table 1**) and constructed into the PX459M and EZ-guide plasmid with *Bbs* I. Then, the gRNA2 was subcloned into PX459M-gRNA1 by *Xho* I and *Hin*d III. The plasmid with two gRNA named PX459-gRNA1/2 was used to transfect Marc-145 cells. At 36 hpt, the cells were passaged and diluted into a six-well plate with the media to which puromycin (15 μg/ml) was added. The cells were maintained in selective media for 48 h. Then, the clones were separated into a 96-well plate with single or double cells in each hole. The media was refreshed the following day, and clones were isolated after single-cell plating. Following PCR amplification of a 500-bp region centered on the CRISPR guide, indels were analyzed by sequencing. A WB using a specific cGAS antibody (HPA031700; Sigma, USA) was performed to confirm the absence of the target protein.

### Confocal Fluorescence Microscopy

Marc-145 cells were infected or uninfected with PRRSV at MOI = 0.5. Then, 24 hpt, the cells were fixed in 4% paraformaldehyde for 30 min, blocked with 3% bovine serum albumin for 1 h and permeabilized with 0.1% Triton X-100 for 15 min. The transfected cells were incubated with the indicated antibodies for 1 h at 37°C and washed thrice with PBS. The cells were then incubated at 37°C for 1 h with secondary antibodies, then stained with 1 g/ml of 4, 6-diamidino-2-phenylindole (DAPI) for 10 min, and examined using a Zeiss confocal system.

### Electron Microscopy

Marc-145 cells were seeded into a 5-cm dish and infected with PRRSV. At 36 hpi, the supernatant was discarded and washed thrice with PBS. The cells were fixed at 2% glutaraldehyde. Cell pellets were embedded in 2% agarose, postfixed with 1% osmium tetroxide, and dehydrated with an ethanol series. Samples were infiltrated, embedded, and polymerized for 48 h at 60°C. Ultrathin sections were prepared and examined using a transmission electron microscope (20). The uninfected Marc-145 cells act as the negative control.

### cGAMP Activity Assay

Marc-145 cells were transfected for 30 h with p3xFlag-cGAS or control plasmid before being infected with or without PRRSV at an MOI of 0.5 for 36 h. To test for cGAMP activity, cells were lysed with hypotonic buffer (10 mM Tris·HCl, pH 7.4, 10 mM KCl, 1.5 mM MgCl_2_) and centrifuged at 100,000×*g* for 20 min. The supernatants were incubated at 95°C for 5 min before being centrifuged at 12,000×*g* for 5 min. Supernatants were recovered and treated or 30 min at 37°C with 1 U/ul of benzonase (EMD Millipore), followed by 60 min at 50°C with 100 μg/ml of Proteinase K (Thermo Fisher Scientific). The supernatant was incubated for 5 min at 95°C before being cooled to 25°C. The supernatant was treated with 1× digitonin permeabilization solution. The buffer was incubated with Marc-145 cells for 20 min at 37°C. The digitonin permeabilization solution was aspirated from the cells and replaced with normal growth media. RNA was isolated from the Marc-145 cells 12 h later, and the IFN transcript was analyzed (21).

### Statistical Analysis

All the experiments mentioned above were performed using three independent experiments. Statistical significance was analyzed using t-tests. The data shown are the means ± standard variations (SD) of three independent experiments. P-values of less than 0.05 were considered statistically significant.

## Results

### Overexpression of cGAS Induces the Production of IFN-β During PRRSV Infection

**Figure 1:**
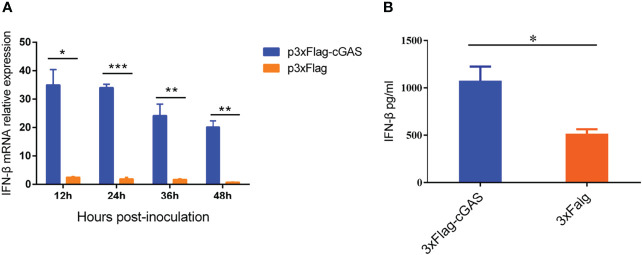
cGAS increases IFN-β expression during PRRSV infection. **(A)** IFN-β relative mRNA level in Marc-145 cells transfected with p3XFLAG or p3XFLAG-cGAS after being infected with PRRSV. **(B)** The Marc-145 cells were transfected with p3XFLAG or p3XFLAG-cGAS for 36 h and then infected with PRRSV, the IFN-β production was detected by Monkey Interferon β ELISA Kit. The statistical significance of differences was determined using Student’s *t-*test (*p <0.05; **p <0.01; ***p <0.001).

As a DNA sensor, cGAS can be activated to induce the production of IFN-β and ISGs during DNA virus infection. However, our findings showed that an RNA virus overexpressing the cGAS during PRRSV infection could also promote IFN-β production at the transcription and protein levels. As shown in **Figure 1A**, overexpression of cGAS significantly increases the IFN-β at the mRNA level during PRRSV infection. Furthermore, at 12 hpi, the content of IFN-β in the supernatant of the Marc-145 transfected with cGAS was significantly higher than that of untransfected cGAS (*p <*0.05) (**Figure 1B**). cGAS induces the production of IFN-β during PRRSV infection, according to all of these studies.

### cGAS Exhibits Anti-PRRSV Activity

**Figure 2:**
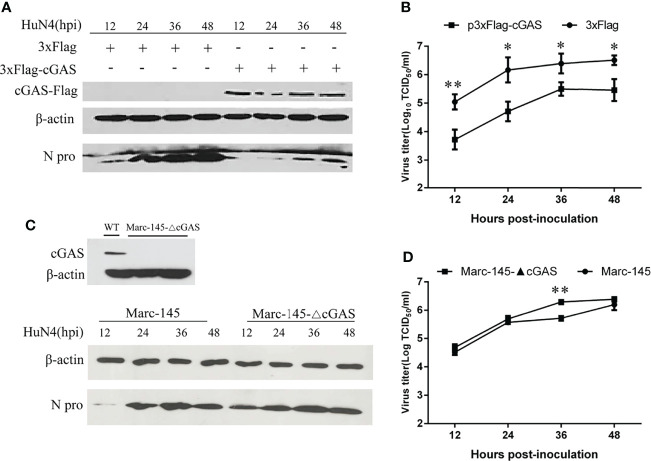
cGAS affects the replication of PRRSV. Marc-145 cells transfected with p3×Flag-cGAS (2 μg) or p3×Flag (2 μg) were inoculated with the PRRSV HuN4 strain at an MOI of 0.1. The cells and supernatant were harvested at the indicated times. **(A)** The replication of PRRSV was evaluated by WB with an anti-N polyclonal antibody. **(B)** The infectious viral titer in the supernatant was determined by a microtitration assay and calculated as log_10_ TCID_50_ per ml. **(C)** WB analysis of endogenous cGAS levels in cGAS-KO Marc-145 cells. cGAS-KO Marc-145 cells and wild-type Marc-145 cells were infected with PRRSV at an MOI of 0.1. The replication of PRRSV was evaluated by WB with an anti-N polyclonal antibody. **(D)** The viral titer in the supernatant was determined by TCID_50_. β-actin expression was used as a loading control. The data are presented as the mean ± SD from three experiments. The statistical significance of differences was determined using the Student’s *t*-test (*p <0.05; **p <0.01).

To examine the effects of cGAS on PRRSV replication, cGAS was overexpressed in the Marc-145 cells by transfection with p3xFlag-cGAS. As shown in **Figure 2A**, when cGAS was overexpressed, the N protein levels of PRRSV were lower than those in the control cells, especially at 12–36 hpi. Furthermore, there was a significant difference in viral titers between cells transfected with p3xFlag-cGAS or p3xFlag, with an approximate 1.0–2.0 log decrease in virus titers from 12 to 36 hpi (*p <*0.05, *p <*0.01) (**Figure 2B**). In contrast, when the cGAS gene was knocked out in the Marc-145 cells, the N protein levels of PRRSV were higher than those in wild-type Marc-145 cells (**Figure 2C**). The virus titers were increased during PRRSV infection at different times and were significantly different in the cGAS knockout Marc-145 cells at 36 hpi (*p <*0.01). These observations suggest that cGAS is a cellular antiviral factor that represses PRRSV infection.

### Mitochondria Were Damaged During PRRSV Infection

**Figure 3:**
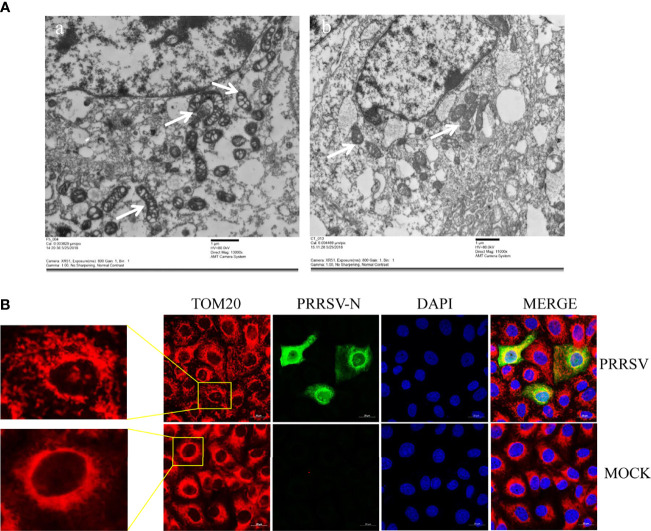
PRRSV infection induces mitochondrial damage. **(A)** Electron microscopy analysis showing mitochondrial fission and defects in PRRSV-infected cells (Left). At 24 h post-infection, PRRSV infected Marc-145 cells, and uninfected cells were examined by electron microscope. The images show elongated mitochondria in uninfected cells and fragmented mitochondria with defective cristae in infected cells (White Arrow). **(B)** At 24 h post-infection, Marc-145 cells were immunostained with antibodies specific to TOM20 (red), N (green). The zoomed images show typical tubular mitochondria in uninfected cells and fragmented mitochondria in PRRSV infected cells.

Mitochondria are key participants in innate immune pathways, functioning as both signaling platforms and contributing to effector responses. mtDNA, engaging PRRs and triggering type I IFNs and ISG expression, may leak into the cytoplasm when mitochondria are damaged. To investigate the morphological alteration of mitochondria after PRRSV infection, Marc-145 cells were infected with PRRSV at an MOI of 0.5 and were subjected to IFA and transmission electron microscopy analysis. The results showed that TOM20, the mitochondrial protein, was typically tubular in uninfected cells and fragmented in PRRSV infected cells (**Figure 3A**). Besides, transmission electron microscopy results showed that the mitochondria were fragmented, and the number of mitochondrial ridges was significantly reduced. Furthermore, the mitochondrial morphology appears vacuolar when infected with PRRSV (**Figure 3B**). Together, these results demonstrate that PRRSV infection induces mitochondrial damage.

### mtDNA Leak to the Cytoplasm During PRRSV Infection

**Figure 4:**
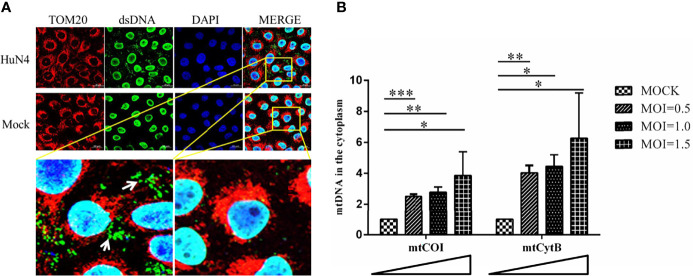
PRRSV infection induces mtDNA leaking into the cytoplasm. **(A)** At 24 h post-infection, Marc-145 cells were immunostained with antibodies specific to TOM20 (red), dsDNA (green). The nucleus is stained with DAPI (blue) in the merged images. The zoomed images show that large amounts of dsDNA (green) accumulate in the cytoplasm of the PRRSV infected cells. **(B)** Fold induction of levels of mitochondria-specific DNA sequences mtCOI and mtCytB present in the cytosol of Marc-145 cells infected with PRRSV at MOI 0.5, 1 and MOI 1.5. The statistical significance of differences was determined using the Student’s *t*-test (*p <0.05; **p <0.01; ***p <0.001).

As PRRSV infection induces mitochondrial damage, we hypothesize the mtDNA is leaked to the cytoplasm when there is a PRRSV infection. To further verify this hypothesis, the Marc-145 cells infected with or without PRRSV were subjected to IFA and qPCR to detect the mtDNA in the cytoplasm. As shown in **Figure 4A**, there is a large amount of double-stranded DNA (dsDNA) in the cytoplasm of PRRSV-infected cells, but there is no double-stranded DNA in the cytoplasm of uninfected cells. Besides, qPCR results showed that the amount of mtDNA was significantly increased when PRRSV infection was detected (**Figure 4B**; p <0.05, p <0.01, p <0.001). Moreover, as the dose of infection increases, the amount of mtDNA in the cytoplasm also increases. These results suggest that mtDNA is leaked into the cytoplasm when there is a PRRSV infection.

### mtDNA Co-Location With cGAS During PRRSV Infection

**Figure 5:**
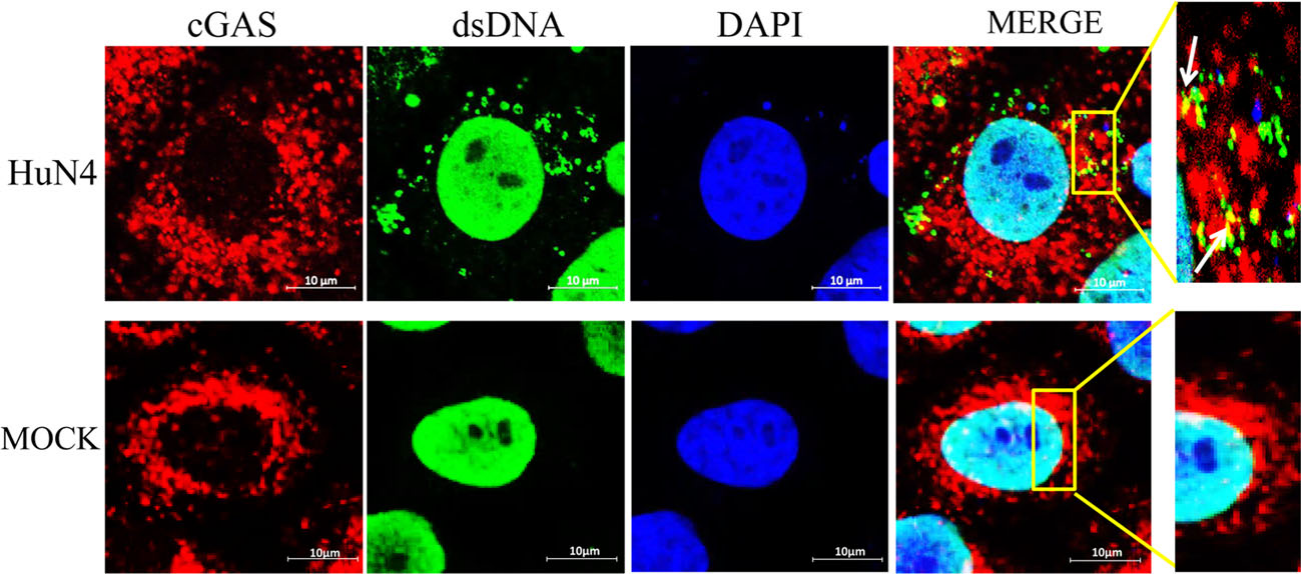
mtDNA co-located with cGAS during PRRSV infection. Marc-145 cells were infected with the PRRSV HuN4 strain and the uninfected served as the negative control. The cells were fixed 24 h after infection and then subjected to indirect immunofluorescence to detect cGAS protein (red) and mtDNA (green). The nucleus is stained with DAPI (blue). The zoomed images show that mtDNA co-located with cGAS in the infected Marc-145 cells (yellow).

To confirm whether the cytoplasmic mtDNA induced by PRRSV infection can bind to cGAS, the Marc-145 cells infected with PRRSV were fixed to verify co-localization between cGAS and mtDNA. The results showed that PRRSV infection could induce mtDNA into the cytoplasm and co-localize with cGAS (**Figure 5**).

### cGAS Activates cGAMP Activity During PRRSV Infection

**Figure 6:**
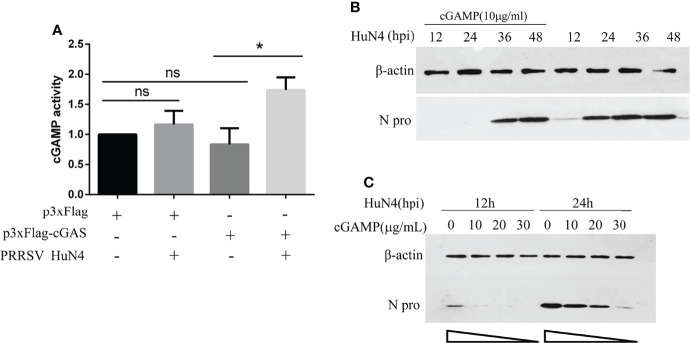
cGAS activates cGAMP activity during PRRSV infection, and cGAMP inhibits PRRSV replication. **(A)** Marc-145 cells were transfected with p3xFlag, p3xF-cGAS, and infected with or without PRRSV. The cGAMP activity was measured by qRT-PCR to detect the transcript levels of IFNb1. The statistical significance of differences was determined using the Student’s *t*-test (*p <0.05; ns, not significant). **(B)** The Marc-145 cells were treated with cGAMP for 12 h at different concentrations and then infected with PRRSV at 0.5 MOI. The cells were harvested at 12 and 24 hpi. The N protein level was detected by WB with anti-N pAb. **(C)** The Marc-145 cells were treated with cGAMP of 10 μg/ml for 12 h, and the cells were infected with PRRSV at 0.5 MOI. The cells were harvested at a different time, and the N protein was detected by WB with the anti-N pAb.

We have demonstrated that PRRSV replication can be inhibited by cGAS. Besides, the cGAS, acting as the viral sensor, can activate the cGAMP. So, we detected cGAMP activity during PRRSV infection. The findings show that cGAS overexpressing alone cannot increase cGAMP activity, and PRRSV infection alone can only increase cGAMP activity to a certain extent. The cGAMP activity was significantly increased when overexpressing cGAS during PRRSV infection (**Figure 6A**). This phenomenon may be because PRRSV infection causes mtDNA to leak into the cytoplasm, which binds to cGAS and then activates cGAMP activity. While overexpressing cGAS alone does not increase cGAMP activity because there is no mtDNA as an activator.

### PRRSV Was Restricted by cGAMP

cGAS inhibits PRRSV replication, and cGAS can activate the activity of cGAMP. To test whether the PRRSV was restricted by cGAMP, Marc-145 cells were treated with cGAMP at 10 μg/ml. The N protein level of PRRSV was significantly decreased at different times (**Figure 6B**). Additionally, the Marc-145 cells were treated with different concentrations of cGAMP and then infected with PRRSV. The results indicated that the expression level of N protein was decreased with increasing cGAMP concentration (**Figure 6C**). The results demonstrated that PRRSV was dose-dependently restricted by cGAMP.

### cGAS Suppresses Different Genotypes of PRRSV

To verify the inhibition of cGAS on classic type 2 (vAPRRS) and classic type 1 strains (vSHE), the Marc-145 cells were transfected with cGAS and then infected with the vAPRRS and vSHE strains. The results showed that the viral titer and the N protein level of the virus were significantly decreased compared with the untransfected with the cGAS. The results indicated that cGAS could restrict multiple PRRSV strains.

## Discussion

PRRSV has been a major threat to global industrial pig farming since its discovery in the late 1980s (22), particularly during the HP-PRRSV outbreak in 2006 (23). Vaccination has been used to control PRRSV. However, commercially available vaccines fail to provide sustainable protection against PRRSV due to its rapid evolution. Antiviral therapies provide creative insights to guide future PRRSV control and prevention efforts, especially in cases where existing vaccines fail to match the circulating virus (24). Our study found that overexpression of cGAS inhibits PRRSV replication, while knockout cGAS facilitates PRRSV replication in Marc-145 cells (**Figure 2**). During PRRSV infection, cGAS can activate cGAMP activity and then induce the production of IFN to inhibit PRRSV replication (**Figures 1**, **6**). Our results showed that cGAS, as a newly discovered ISG, might have potential use as a novel approach to control PRRSV infection. Therefore, understanding the mechanism of cGAS restriction of PRRSV will be beneficial for developing effective therapies to control outbreaks of this disease in the future.

As the first line of host defense, the innate immune system uses PRRs to detect invading pathogens. cGAS is a conserved component of the innate system in DNA virus infection. It binds to cytosolic double-stranded DNA (dsDNA) from various sources, including bacteria, DNA viruses, and retroviruses. Following the binding of dsDNA, cGAS catalyzes the production of a second messenger known as cGAMP, which subsequently binds to the adaptor protein STING on the endoplasmic reticulum (ER) membrane (25). STING recruits TANK binding kinase 1 (TBK1) and activates transcription factors interferon regulatory factor 3 (IRF3) and nuclear factor-κB (NF-κB), which then translocate into the nucleus to induce the production of IFN and other inflammatory cytokines, establishing an antiviral state in infected and uninfected neighboring host cells (26). cGAS inhibited the replication of many DNA viruses, including HSV-1, MHV68, and vaccinia virus. Currently, this is considered the classical pathway by which cGAS exerts its antiviral effect (13). Several studies have revealed that cGAS is also engaged in antiviral responses to RNA viruses, namely, EAV, DENV, WNV, IAV, and CHIKV (14–16). However, how cGAS is involved in RNA virus-induced immune responses is largely unknown. Although cGAS was reported to bind dsRNA, this interaction did not lead to the production of cGAMP (27). Therefore, cGAS may play its role in antiviral responses to RNA viruses differently from the classical pathway.

Our study demonstrated that the cGAS is important in inhibiting PRRSV replication, consistent with previous studies (28). Their study suggested that cGAS and STING possess the anti-PRRSV effect. Nevertheless, the mechanism is unclear (28). Our findings show that cGAS overexpression promotes IFN-β production during PRRSV infection at both the transcription and translation levels (**Figure 1**). Additionally, overexpression of cGAS decreased the N protein level of PRRSV and the viral titer.

In contrast, knockout cGAS facilitates the replication of PRRSV (**Figures 2C, D**). While PRRSV has been identified and characterized as an IFN-β antagonist virus, the Nsp1, Nsp2, Nsp4, Nsp11, and N protein of PRRSV play an important role in its innate immunity antagonism (29). Besides, during PRRSV infection, the virus genome was released into the cytoplasm, ORF1a and ORF1b were translated to produce two large polyproteins and autocatalytic processing to generate at least 14 Nsps. Some Nsps assemble into a replication and transcription complex (RTC) to direct genome amplification and subgenomic mRNA synthesis to produce a nested set of six major subgenomic mRNAs that are both 5’- and 3’-coterminal with the genomic RNA (30). No DNA or DNA intermediate exists in the life cycle of PRRSV. Even so, cGAS can still activate innate immunity and exert anti-PRRSV effects, so we hypothesized that cGAS exerts antiviral effects through a non-canonical pathway.

To further elucidate the molecular mechanism by which cGAS activates innate immunity to suppress PRRSV replication, we examine the effects of PRRSV infection on mitochondria. The results suggest that PRRSV infection-induced mitochondrion damage and leaked mtDNA into the cytoplasm are dose-dependent (**Figure 4B**). When mtDNA is released into the cytoplasm, it can act as a danger-associated molecular pattern (DAMP) to stimulate IFN-β production *via* the cGAS–STING pathway (31). cGAS catalyzes the production of a second messenger known as cGAMP, which subsequently binds to the adaptor protein STING on the endoplasmic reticulum (ER) membrane to activate innate immunity (25). Our findings show that cGAS overexpression alone cannot increase cGAMP activity and PRRSV infection alone can only increase cGAMP activity to a certain extent.

**Figure 7:**
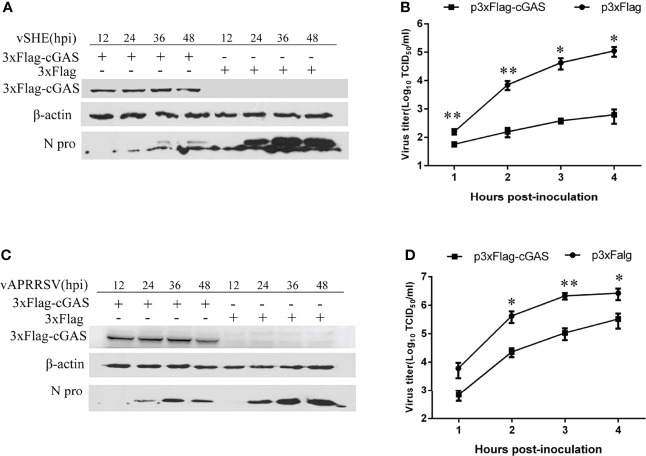
cGAS overexpression inhibits classical Europe-type (genotype I) and North America-type (genotype II) PRRSV replication. **(A, B)** PRRSV vSHE strain (genotype I) and **(C, D)** vAPRRSV (genotype II) strain proliferation in MARC-145 cells transfected with p3xFlag-cGAS or control plasmids. The N protein expression levels of PRRSV were analyzed by WB. Viral titers from Marc-145 cells transfected with p3xFlag-cGAS or control plasmids were determined by a microtitration assay and calculated as log_10_ TCID_50_ per ml. Data represent the mean ± standard deviation of three independent experiments. Statistical significance was analyzed using Student’s t-test (*p <0.05; **p <0.01).

In contrast, the cGAMP activity was significantly increased upon cGAS being overexpressed during PRRSV infection. Therefore, the increase in cGAMP activity depends on two necessary conditions, PRRSV infection and cGAS (**Figure 6A**). Only during PRRSV infection can mtDNA be released into the cytoplasm to activate cGAS and then catalyze the production of cGAMP. cGAMP can bind to the adaptor protein STING and then coordinate the activation of inflammatory transcription factors to induce IFN expression and establish an antiviral cellular state (32). Besides, laser confocal results confirmed that cGAS can co-localize with mtDNA in the cytoplasm during PRRSV infection. Furthermore, Marc-145 cells treated with cGAMP alone can inhibit the PRRSV (**Figures 6B, C**). Moreover, besides HP-PRRSV, cGAS suppresses the classic type 1 strain (vSHE) (**Figures 7A, B**) and the classic type 2 strain (vAPRRS) (**Figures 7C, D**). Compared with the type 2 PRRSV virus, cGAS had a stronger inhibitory effect on type 1 PRRSV, which may be more sensitive to cGMAP. These results elucidate that cGAS inhibits multiple PRRSV replication. Besides PRRSV, DENV was also restricted by cGAS with a similar method (19, 33, 34). Certainly, RNA viruses have evolved effective strategies to antagonize the function of cGAS to facilitate their replication in host cells. DENV NS2B protease cofactor targets the cGAS for lysosomal degradation to avoid the detection of mtDNA during infection (34). CHIKV inhibits type-I interferon responses mediated by cGAS-STING by degrading cGAS (16). Virus and cGAS are always in a state of mutual antagonism, but how PRRSV antagonizes the antiviral effect of cGAS in natural infection remains to be further studied.

Overall, this study demonstrated that PRRSV infection induces mitochondrial damage and leaks mtDNA into the cytoplasm. cGAS restricts PRRSV replication by detecting the mtDNA in the cytoplasm, activating the cGAMP activity, and inducing the production of IFN-β. These results establish a foundation for further exploration of cGAS involved in resistance to PRRS transmission. Our study highlights the importance of cGAS in PRRSV replication and suggests potential antiviral therapies.

## Data Availability Statement

The original contributions presented in the study are included in the article/supplementary material. Further inquiries can be directed to the corresponding author.

## Author Contributions

KZ designed the experiments. KZ, XL, LL, FG, YJ, GL, and LY performed the experiments. KZ and LL analyzed the data and wrote the manuscript. WY, YZ, and GT made constructive comments on the experiments. All authors listed have made a substantial, direct, and intellectual contribution to the work and approved it for publication.

## Funding

This work was supported by the National Natural Science Foundation of China (Grant No. 32102644); the Talents Introduction Projects of Hebei Agricultural University (Grant No. YJ201945); the Key Research and Development Projects of Hebei (Grant No. 20326625D); the State Key Laboratory of Veterinary Etiological Biology, Lanzhou Veterinary Research Institute, Chinese Academy of Agricultural Sciences (Grant No. SKLVEB2020KFKT016); Basic Scientific Research Funds of Provincial Universities in Hebei Province (KY202017).

## Conflict of Interest

The authors declare that the research was conducted in the absence of any commercial or financial relationships that could be construed as a potential conflict of interest.

## Publisher’s Note

All claims expressed in this article are solely those of the authors and do not necessarily represent those of their affiliated organizations, or those of the publisher, the editors and the reviewers. Any product that may be evaluated in this article, or claim that may be made by its manufacturer, is not guaranteed or endorsed by the publisher.
